# Supplementary material for: Representing Structural Isomer Effects in a Coarse-Grain Model of Poly(Ether Ketone Ketone)
Source: Polymers (Basel). 2025 Jan 5;17(1):117. doi: 10.3390/polym17010117 (PMC11722673; doi:10.3390/polym17010117)
Supplement: Supplementary file 1 [file polymers-17-00117-s001.zip › polymers-3398952-supplementary-materials.pdf]

Supplementary Materials: REPRESENTING STRUCTURAL ISOMER EFFECTS IN A COARSE-GRAIN MODEL OF POLY(ETHER KETONE KETONE)

Chris D. Jones [1], Jenny W. Fothergill [1], Rainier Barrett [1], Lina N. Ghanbari [2], Nicholas R. Enos [2], Olivia McNair [2], Jeffrey Wiggins [2], Eric Jankowski [1,*]

1. Mathematical Expressions and Nomenclature

Below is a concise summary of the mathematical expressions used in the main text, and the definition of their variables. Equations are listed in the order that they appear in the main text.

Boltzmann Inverse:

$$V(x) = -k_b T \ln[P(x)] \tag{1}$$

The Boltzmann inverse is used to obtain the potential of mean force form a corresponding probability distribution.

- x: Independent variable (e.g., pair distance, bond angle, etc.)
- $V(x)$: The potential of mean force
- k_b: Boltzmann's constant
- T: Temperature
- $P(x)$: Probability distribution

Harmonic Bond Force:

$$V_{bond}(l) = \frac{1}{2}k(l - l_0)^2 \tag{2}$$

This is the functional form used in the coarse-grain model for calculating harmonic bond energies.

- l: Bond length
- $V(l)$: Potential energy as a function of bond length
- k: Force constant
- l_0: Equilibrium bond length

Periodic Dihedral Force:

$$V_{dihedral}(\phi) = \frac{1}{2}k(1 + d\cos(\phi - \phi_0)) \tag{3}$$

This is the functional form used in the coarse-grain model for calculating periodic dihedral energies.

- ϕ: Dihedral angle
- $V(\phi)$: Potential energy as a function of dihedral angle.
- k: Force constant
- ϕ_0: Equilibrium dihedral angle
- d: Sign factor

Multi-state Iterative Boltzmann Inversion Potential Update:

$$V_{i+1}(x) = V_i(x) - \frac{1}{N}\sum_s \alpha_s k_B T_s \ln\left[\frac{P_s^i(x)}{P_s^*(x)}\right] \quad (4)$$

This defines how tabular potentials are updated after each iteration during MSIBI optimization[1].

- x: Independent variable (e.g., pair distance, bond angle, etc.)
- i: Iteration number
- $V_i(x)$: Potential of iteration i
- $V_{i+1}(x)$: Potential of next iteration $(i+1)$
- N: Number of state points
- s: State point
- α_s: State point weighting factor
- k_B: Boltzmann's contant
- T_s: State point temperature
- $P_s^i(x)$: State point probability distribution resulting from iteration i
- $P_s^*(x)$: State point target probability distribution

Curve Fitting Score

$$f_{fit} = 1 - \frac{\sum_{x_{start}}^{x_{cut}}\left(|P^i(x) - P^*(x)|\right)}{\sum_{x_{start}}^{x_{cut}}\left(|P^i(x)| + |P^*(x)|\right)} \quad (5)$$

This is used to provide a quantitative match between two probability distributions[1].

- f_{fit}: Score ranging from 0 to 1
- x: Independent variable (e.g., pair distance, bond angle, etc.)
- i: Iteration number
- $P^i(x)$: Probability distribution resulting from iteration i
- $P^*(x)$: Target probability distribution

2. MSIBI State Points

When using multi-state iterative Boltzmann inversion (MSIBI) we have control over which fine-grained state points to learn from. These state point choices are something that can be enumerated over and optimized if desired. In this work, we started by choosing state points that cover the relatively small range of densities and temperatures relevant to modeling fusion welding of PEKK interfaces. This includes covering the temperature range of below glass transition (T_g) to just above melting (T_m), and densities including amorphous ($1.27\frac{g}{cm^3}$) to crystalline ($1.38\frac{g}{cm^3}$)[2]. The initial set of state points used in developing pair potentials are highlighted in Table S1. The pair potentials created from these state points resulted in good RDF matching for each pair at all state points. However, when comparing the coarse-model against target chain dimensions of squared radius of gyration ($\langle R_g^2\rangle$) and squared end-to-end distance ($\langle R_e^2\rangle$) this coarse-model did not successfully re-create target values.

We re-visited the development of the pair potentials while replacing the original State A of amorphous density and T below T_g with a two-chain low density state. The other 3 bulk state points remained the same as shown in Table S2. The pair potentials developed from the second set of state points resulted in similar RDF matching f_{fit} scores,

Table S1. Initial target state points used in MSIBI

State	Temperature C°	Density $\frac{g}{cm^3}$
A	20	1.38
B	255	1.27
C	255	1.35
D	414	1.27

Table S2. Final target state points used in MSIBI

State	Temperature C°	Density $\frac{g}{cm^3}$
A	414	0.0003
B	255	1.27
C	255	1.35
D	414	1.27

but performed much better in matching the target squared radius of gyration $(< R_g^2 >)$ and the squared end-to-end distance $(< R_e^2 >)$ as shown in Figure S1.

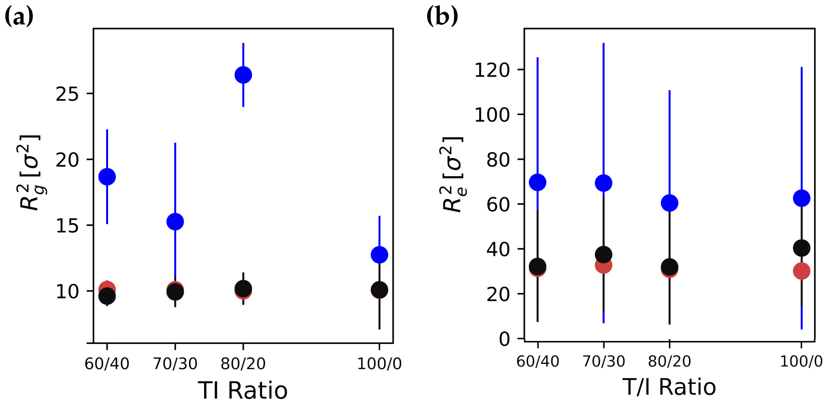

Figure S1. Comparison of a) R_g^2 and b) R_e^2 between target UA model (black), CG model trained with the state points in Table S1 (blue) and CG model trained with state points in Table S2 (red). The addition of the low density two-chain state (State A in Table S2) results in significant improvement.

3. IBI and MSIBI Distribution Matching

In this section, we show detailed figures of RDF comparison between UA and CG models, as well as the complete set of f_{fit} scores for the coarse-grain model. As described in the Methods section of the main text f_{fit} gives the quantitative match between two distributions and is given by Equation 5 [1]. Detailed views of RDF comparisons across all four state points are shown in Figure S2 for E−E pairs, Figure S3 for E−K pairs and Figure S4 for K−K pairs.

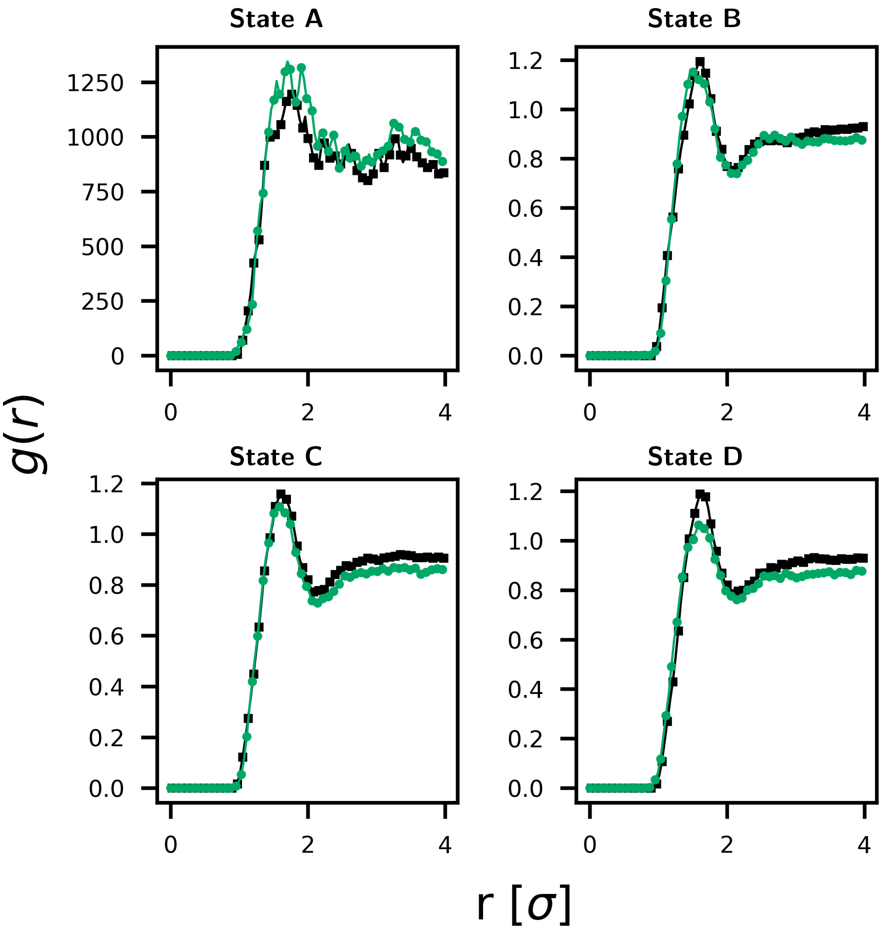

Figure S2. Comparison between RDFs for E−E pairs. Target UA model are shown with black lines, and the CG model with colored lines.

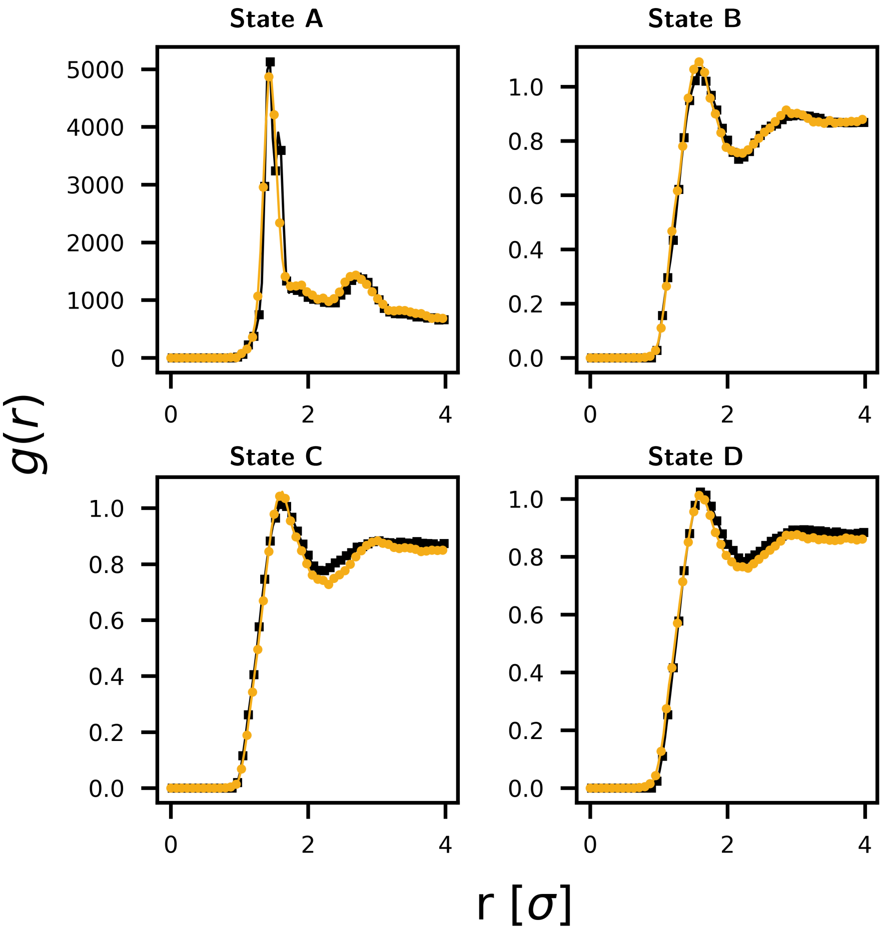

Figure S3. Comparison between RDFs for E−K pairs. Target UA model are shown with black lines, and the CG model with colored lines.

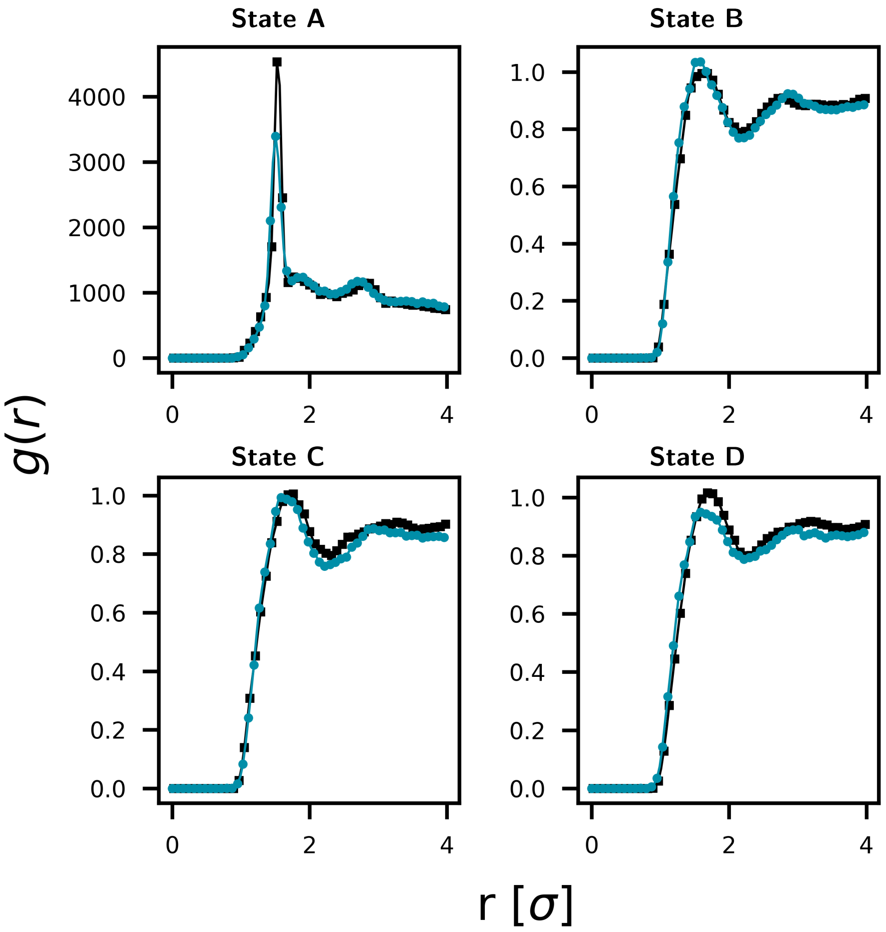

Figure S4. Comparison between RDFs for K−K pairs. Target UA model are shown with black lines, and the CG model with colored lines.

The f_{fit} scores for each pair and statepoint (Table S2) are summarized in Table S3. Across all pairs and states, the f_{fit} scores are relatively close to 1.0, especially for the bulk state points (B, C, D). Table S4 shows the f_{fit} scores for the angle distributions at each T/I ratio.

Table S3. RDF f_{fit} scores for all pairs at each state point.

State	Pair Type	Score
A	E−E	0.953
A	K−K	0.954
A	E−K	0.945
B	E−E	0.973
B	K−K	0.982
B	E−K	0.981
C	E−E	0.980
C	K−K	0.989
C	E−K	0.991
D	E−E	0.972
D	K−K	0.981
D	E−K	0.984

Table S4. Angle distribution f_{fit} scores for E−K−K and K−E−K angles at all T/I ratios studied.

T/I Ratio	E−K−K Score	K−E−K Score
60/40	0.987	0.977
70/30	0.986	0.984
80/20	0.980	0.976
100/0	0.985	0.984

4. Chain Statistics Analysis

Here, we report the results of a two-sided *t-test* performed on the data used to compare R_g^2 (Table S5), R_e^2 (Table S6) and ℓ_p (Table S7) as shown in Figure 7 and Figure 8 in the main text. While *p-values*$<$ 0.05 are conventionally used to indicate whether an effect is significant, they do not indicate the size, or practical importance of the effect, so in addition to the *p-value*, we report the effect size using the Cohen's D method, which is a measure of the substantive significance[3]. Heuristics typically used for D are values of $D > 0.8$ are large, $0.2 < D < 0.8$ are medium and $D < 0.2$ are small[3]. For R_g^2 and R_e^2, we mostly see that differences between models are either not statistically significant, or the size of the effect is small to medium. For ℓ_p, we mostly observe both significant differences and large effect sizes, indicating the CG model is less effective at predicting persistence length than radius of gyration and end-to-end distance. However, as shown in Figure 7a and Figure 8a in the main text, the degree to which the CG model over-predicts persistence length is on the order of 0.1σ or 0.34 angstroms.

Table S5. R_g^2 statistical comparison between UA and CG models.

T/I Ratio	T	$\lvert t\,stat \rvert$	$\lvert p\,value \rvert$	$\lvert D \rvert$
60/40	5.0	0.580	0.564	0.150
70/30	5.0	2.217	0.031	0.573
80/20	5.0	2.065	0.043	0.533
100/0	5.0	1.003	0.320	0.259
80/20	4.0	4.181	9.94e-5	1.079
80/20	4.5	0.720	0.475	0.186
80/20	5.5	2.103	0.040	0.543
80/20	6.0	1.138	0.172	0.357

Table S6. R_e^2 statistical comparison between UA and CG models.

T/I Ratio	T	$\lvert t\,stat \rvert$	$\lvert p\,value \rvert$	$\lvert D \rvert$
60/40	5.0	0.987	0.328	0.255
70/30	5.0	2.536	0.0139	0.655
80/20	5.0	1.263	0.212	0.326
100/0	5.0	0.885	0.380	0.229
80/20	4.0	2.579	0.0125	0.666
80/20	4.5	0.477	0.635	0.124
80/20	5.5	3.982	7.60e-5	0.412
80/20	6.0	3.888	1.139e-4	0.436

Table S7. ℓ_p statistical comparison between UA and CG models.

T/I Ratio	T	$\lvert t\,stat \rvert$	$\lvert p\,value \rvert$	$\lvert D \rvert$
60/40	5.0	6.627	1.019e-7	2.149
70/30	5.0	1.937	0.061	0.629
80/20	5.0	2.974	0.005	0.965
100/0	5.0	2.052	0.048	0.666
80/20	4.0	5.757	1.470e-5	1.868
80/20	4.5	4.967	1.660e-5	1.611
80/20	5.5	0.355	0.725	0.115
80/20	6.0	2.116	0.041	0.686

References

1. Moore, T.C.; Iacovella, C.R.; McCabe, C. Derivation of coarse-grained potentials via multistate iterative Boltzmann inversion. *The Journal of Chemical Physics* **2014**, *140*, 224104. https://doi.org/10.1063/1.4880555.
2. Tencé-Girault, S.; Quibel, J.; Cherri, A.; Roland, S.; Fayolle, B.; Bizet, S.; Iliopoulos, I. Quantitative Structural Study of Cold-Crystallized PEKK. *ACS Applied Polymer Materials* **2021**, *3*, 1795–1808. https://doi.org/10.1021/acsapm.0c01380.
3. Sullivan, G.M.; Feinn, R. Using Effect Size—or Why the *P* Value Is Not Enough. *Journal of Graduate Medical Education* **2012**, *4*, 279–282. https://doi.org/10.4300/JGME-D-12-00156.1.
